# A descriptive and comparative analysis of injuries reported in USA Cycling-sanctioned competitive road cycling events

**DOI:** 10.1186/s40621-022-00385-7

**Published:** 2022-07-14

**Authors:** Gregory Jancaitis, Alison R. Snyder Valier, Curt Bay

**Affiliations:** 1 Norwich University, 158 Harmon Drive, Northfield, VT 05663 USA; 2 A.T. Still University, 5850 E. Still Circle, Mesa, AZ 85206 USA

**Keywords:** Bicycling, Competition, Emergency medicine, Acute injury

## Abstract

**Background:**

Competition in road cycling events is common, yet little is known about the nature and disposition of injuries sustained in these events. The purpose of this study is to describe injured body regions and the disposition of injuries sustained by cyclists during competitive road cycling events.

**Methods:**

Data regarding body region injured and injury disposition were retrospectively analyzed from a convenience sample of 1053 injury reports (male: *n* = 650 [61.7%], age = 33.4 ± 13.6 years; female: *n* = 116 [11.0%], age = 33.3 ± 13.9 years; missing: *n* = 284 [27.0%]) completed during the 2016 competitive season.

**Results:**

A total of 1808 injuries were reported. Injured body regions included upper extremity (46.5%, *n* = 841), lower extremity (32.2%, *n* = 583), head/neck (10.4%, *n* = 189), torso/back (5.2%, *n* = 95), face (4%, *n* = 87), and internal/other (0.7%, *n* = 13). There were 1.37 ± 0.81 injuries recorded per report. Dispositions following injury were medical attention (34.1%, *n* = 316), ambulance/EMS (19.3%, *n* = 179), report only (15% *n* = 139), referred (13.0% *n* = 121), released to parent/personal vehicle (12.1% *n* = 112), refused care (4.1% *n* = 38), and continued riding (2.5% *n* = 23). Males (34.0%, *n* = 212) received medical attention more frequently than females (23.3%), *p* < 0.05. Females received EMS transport (29.1%, *n* = 30) more frequently than males (16.8%, *n* = 105), *p* < 0.05.

**Conclusions:**

Upper extremity is the most injured body region in this data set. Following injury, racers often receive medical attention and a substantial percentage require transport by EMS.

**Clinical relevance:**

Anticipating the nature of injuries sustained by cyclists may promote positive health outcomes by ensuring medical teams are prepared for the immediate medical needs of cyclists.

## Background

Participation in cycling events has increased in recent years with multiple formats of competitive and non-competitive events held across the country. The National Governing Body for cycling in the USA, USA Cycling, offers National Championship competitions in 18 different disciplines including on- and off-road events (Cycling 2017). In 2013, USA Cycling licensed more than 64,000 cyclists and sanctioned over 3100 events (Cycling 2013). Non-competitive events such as charity rides often draw thousands of participants of various skill and ability levels. Participation, rather than performance, is often the emphasis. Mountain bike races occur on a variety of non-paved and off-road surfaces and require racers to negotiate a variety of trail obstacles such as rocks, roots, jumps, and steep descents. Competitive road cycling includes a variety of disciplines, including criteriums, single-day and multi-day (stage) races, and time trials. Criteriums involve packs of riders competing on a short, closed course consisting of multiple turns. Single-day and stage races involve packs of riders covering long distances on public roadways that may be closed to traffic, open to traffic, or have rolling road closures. Time trials involve riders competing on a road course individually.

The incidence of injury has been described in non-competitive road cycling events (Boeke et al. 2010; Emond et al. 1999; Roi and Tinti 2014) and in competitive mountain bike events (Kronisch et al. 1996). The variability between non-competition road events, mountain events, and competitive road events limits generalizability of information across disciplines. The literature examining injuries in competitive road cycling is limited to small-scale descriptive studies that surveyed professional male cyclists about injuries throughout their career and do not provide information specific to the risks of road cycling competitions (Barrios et al. 2015). Little is known about the nature of injuries suffered by cyclists participating in road cycling competitions. Specifically, there are limited data on the use of specific medical services, injuries sustained, number of body regions injured, and sex and age differences in injury patterns during competitive road cycling events.

Injury disposition, or the level of care rendered to an injured cyclist, has been previously classified as presenting to an emergency department (Chen et al. 2013), calling for an ambulance or emergency services (Roi and Tinti 2014), and inability to continue participating (Kronisch et al. 1996). Most, if not all, sanctioned cycling events have medical personnel on hand to provide on-site medical services to cyclists or determine when further care is needed. Medical teams are typically multi-disciplinary in nature, incorporating physician, nursing, athletic training, and emergency medical services (EMS) professionals; however, to our knowledge there is no standard for which medical professionals should be involved in on-site care during cycling events. An examination of records from competitive cycling events will provide a more complete picture of the injuries that occur during races and should be evaluated to better understand medical needs and services at race events.

In addition to being prepared to accommodate different injury dispositions, medical teams at competitive cycling events should appreciate the frequency with which different body regions are injured by cyclists. Upper extremity injuries appear to be a common body region reported in emergency department data (Chen et al. 2013) and during non-competitive road cycling and competitive mountain biking events (Boeke et al. 2010; Kronisch et al. 1996). An analysis of competitive road cycling events will help determine if this trend is consistent with non-competitive and mountain bike events and may assist medical teams providing care at competitive events in their preparation for what types of injuries to expect. 

Sex of participants is not uniformly reported in cycling epidemiology and is infrequently analyzed, and data on injuries sustained by females in road competition are absent. This warrants investigation because determining the severity of injuries sustained and medical care provided to both males and females may assist medical teams and race organizations in developing injury prevention and management strategies that meet the needs of all cyclists.

Competitive road cycling events in the USA have not been examined closely on a large scale, and an examination of the disposition of injuries, body regions injured, and sex differences in injuries occurring in these events is lacking. This information may help medical staff develop protocols, anticipate injuries, and allocate resources appropriately in order to properly manage cyclists’ care. Therefore, the purpose of this study is to examine the injury dispositions, body regions injured, and sex differences of cyclists injured in competitive road cycling events.

## Methods

This study was a retrospective analysis of descriptive epidemiology data based on a convenience sample of available records from USA Cycling events from the 2016 competitive cycling season. Approval for this study was granted by the Institutional Review Board at A.T. Still University, Mesa, AZ.

Data for this retrospective review were obtained from incident report forms that were completed at USA Cycling sponsored competitive road cycling events during the 2016 cycling season. In total, data were reviewed from races conducted across 30 states. Neither the total number of events in these states nor the number of unique events resulting in the included incident report forms was available. All reports that met the inclusion criteria were collected by the lead author through digital photography for later analysis. Incident report forms may be completed by a medical provider involved in the delivery of care or by the race official (Cycling 2021). In order to preserve confidentiality of incident reports, all identifiable information including cyclist, event, race official, and medical personnel descriptors was concealed prior to collection.

Incident reports were included if they detailed an injury to a cyclist and were completed during competitive road cycling events which consisted of the disciplines of criteriums, open-course individual road races, closed-course individual road races, rolling-closure individual road races, stage races, and individual time trials. Incident reports were excluded if the injured person was not a cyclist, the cyclist was participating in a mountain, track, cyclo-cross, BMX, or non-competitive event such as a Gran Fondo, clinic, training ride, or camp, the event was non-competitive, or the report indicated that the injury occurred at a time other than during competition.

Data collection consisted of a manual review of incident report forms. Incident information was extracted from the injury report and entered into a spreadsheet for analysis by the lead author. Incident report forms consisted of a list of options under the headers of Body Part Injured, Primary Injury, and Disposition where options could be checked by the person completing the form. In the event of multiple regions reported on one incident report, the data were interpreted as nominal and not reflective of hierarchical order. Body regions were recorded as listed in the incident reports. When necessary, body regions were later grouped as upper extremity (including clavicle), lower extremity, head/neck, torso/back, face, and internal/other. In reports where more than one disposition was selected (e.g., medical attention AND ambulance/EMS), the disposition placing the greater burden on the medical system was selected and entered into the spreadsheet. The form also included a free-text section for describing the incident. Information in the free-text section ranged from describing the mechanism of injury, to providing more detail on disposition, to serving as a place for documenting specifics of medical care provided. Due to the heterogeneity of open-field comments, this information was not included in this analysis. All data collection and extraction were performed solely by the lead author. Codification and combination of certain categories were reached through consensus between authors (GJ and AV).

Descriptive statistics, including means, standard deviations, and frequencies are reported for variables, as appropriate. Chi-square and Bonferroni-corrected binomial tests were used to assess the relationship between disposition and cyclist sex. An analysis of variance, with Tukey’s honestly significant difference (HSD) tests, was used to compare mean age of cyclists by disposition. Many variables were too sparsely distributed to support significance testing. The criterion for statistical significance was alpha < 0.05 (two-tailed). The analysis was completed using SPSS statistical software, version 25 (IBM Corp, Armonk, New York). The total number of events or participants in events were not available, so we were unable to determine injury rates.

## Results

A total of 1053 injury incident reports met the inclusion criteria and were reviewed. Some fields on these incident reports were left blank. Based on 841 reports, the average age of cyclists was 33.2 ± 13.3 years (212 missing). Sex of the cyclist was recorded in 766 of the reports (male: *n* = 650 [84.8%]; female: *n* = 116 [15.1%]). The sub-event type was identified in 986 of the reports. Frequencies for these categories were: criterium (40.1%, *n* = 423), closed course (23.7%, *n* = 250), open course (21.1%, *n* = 223), rolling course (4.6%, *n* = 49), stage event (2.4%, *n* = 26), and time trial (1.4%, *n* = 15).

The disposition of the cyclist was recorded in 928 (88.1%) reports. No medical care was provided in 21.6% (*n* = 200) of cases, which reflects the report only, refused care, and continued riding dispositions. Local medical care was provided in 46.2% (*n* = 428) of cases, which reflects the medical attention and released to parent/personal vehicle dispositions. Advanced medical care was sought in 32.3% (*n* = 300) of cases, which reflects the EMS/ambulance transports as well as referrals to hospital/physician dispositions. The most frequent dispositions following race injury were medical attention (34.1%, *n* = 316) and ambulance/EMS (19.3%, *n* = 179). The least common disposition was continuation of the ride by the cyclist (2.5%, *n* = 23). A complete distribution of disposition frequencies is given in Table 1.

**Table 1:** Frequency of injury disposition following evaluation

Disposition	Total No. (%)
Medical attention	316 (34.1)
Ambulance/EMS	179 (19.3)
Report only	139 (15.0)
Referred to hospital/doctor	121 (13.0)
Released to parent/personal vehicle	112 (12.1)
Refused care	38 (4.1)
Continued riding	23 (2.5)

Three fields were provided on the incident reports to record the body region(s) injured. A total of 1808 injured body regions were documented, with an average of 1.37 ± 0.81 injured regions per report. One injured body region was recorded in 33.5% of reports (*n* = 353), 2 injured body regions in 25.0% of reports (*n* = 264), 3 injured body regions in 29.3% of reports (*n* = 309), and no body region recorded in 12.0% of reports (*n* = 127). Table 2 presents the frequencies of body regions injured. Most frequently, injuries occurred to the upper (46.5%, *n* = 841) and lower extremities (32.2%, *n* = 583). The least frequently recorded body region on an incident report was “internal/other.” (0.7%, *n* = 13).

**Table 2:** Body region injured frequency by data entry field

Body Region	Region 1	Region 2	Region 3	Total
	No.	No.	No.	No. (%)
Upper extremity	455	278	108	841 (46.5)
Lower extremity	335	191	57	583 (32.2)
Head/neck	72	53	64	189 (10.4)
Torso/back	26	20	49	95 (5.0)
Face	30	30	27	87 (4.8)
Internal/other	8	1	4	13 (0.7)
Total	926	573	309	1808 (100)

 Table 3 presents findings related to injury disposition according to cyclist sex. Chi-square and Bonferroni-corrected binomial tests showed that males (34.0%, *n* = 212) received medical attention more frequently than females (23.3%), *p* < 0.05; however, females received ambulance/EMS transport (29.1%, *n* = 30) more frequently than males (16.8%, *n* = 105), *p* < 0.05. No other differences were identified.

**Table 3:** Disposition of treatment by participant sex

Disposition	Sex		
	Female	Male	Total
Medical attention*	24 (23.3)	212 (34.0)	236 (32.5)
Ambulance/EMS*	30 (29.1)	105 (16.8)	135 (18.6)
Report only	21 (20.4)	91 (14.6)	112 (15.4)
Referred to doctor/hospital/clinic	13 (12.6)	89 (14.3)	102 (14.0)
Released to parent/personal vehicle	10 (9.7)	82 (13.1)	92 (12.7)
Refused care	3 (2.9)	28 (4.5)	31 (4.3)
Continued riding	2 (1.9)	17 (2.7)	19 (2.6)

*Significant difference between males and females, *p* < 0.05 (Bonferroni-corrected)

Means (SD) for age by disposition are provided in Table 4. An analysis of variance showed an omnibus effect (*p* = 0.024) for disposition. However, a Tukey HSD test indicated that only “released to parent/personal vehicle” and “refused care” differed significantly, *p* = 0.42, in mean age of cyclist.

**Table 4:** Disposition of treatment by participant age

Disposition (No.)	Age, Mean (SD)
Released to parent/personal vehicle (103)*	29.7 (14.8)
Medical attention (271)	33.0 (12.9)
Report only (125)	33.2 (12.6)
Referred to doctor/hospital/clinic (96)	33.6 (13.6)
Ambulance/EMS (150)	34.7 (13.5)
Continued riding (20)	35.1 (12.1)
Refused care (33)	38.4 (12.9)
Total (798)	

*Based on Tukey’s HSD, age differed significantly for only “Released to Parent/Personal Vehicle” versus “Refused Care,” *p* = .042

**Fig. 1:**
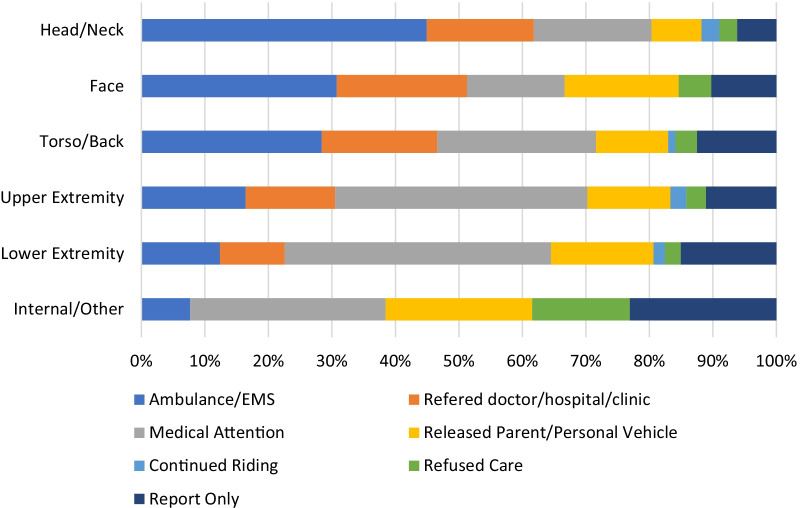
Disposition proportions for each body region injured

Figure 1 provides a breakdown of the various dispositions by region of body injured. As noted above, many reports noted more than one body region per cyclist, so the data should not be interpreted as a one body region-to-one disposition display. It appears, though, that head/neck and facial injuries were most likely to result in ambulance/EMS or referrals to medical establishments. The data were too sparse to be subjected to significance testing.

## Discussion

This study provides the first large-scale descriptive report of frequency of injury disposition, body regions injured, and sex differences in disposition in competitive road cycling events. Prior epidemiologic studies have focused on large-scale non-competitive events, emergency department data, self-report through interview, or a very small-scale, select group of riders (professional cyclists) in and out of competition; hence, there is little epidemiologic data that addresses injuries sustained within competitive road cycling on a large scale and on the scene of the race. These are the first data gathered prospectively and on the scene at cycling events and provide a unique perspective on cycling injuries that differs from other published data on this population of athletes. Through evaluation of incident reports from USA Cycling-sanctioned competitive road cycling events across 30 states, 1053 incident reports were identified. The disposition was presented in 986 of these reports, and there were 1808 body regions injured across the reports.

Criterium racing is a competitive event consisting of multiple laps of a short course taking place over a shorter time duration (generally an hour in length) than most road cycling events. In our data set, criteriums accounted for the largest proportion of injury reports (40.1%, *n* = 423). Our data set did not allow us to assess how many criterium events took place in the 30 states sampled, or how many participants were signed up for each race, so it is not possible in this analysis to determine the true risk of injury based on event type and whether the risk of injury in criteriums is greater than in any other cycling discipline. However, given that 40.1% of injuries reported occurred in criteriums, this style of racing warrants further investigation to determine if there is elevated risk of injury when competing in these events.

Within this sample, some form of medical attention without further medical follow-up was provided in 34% of injury presentations and ambulance/EMS services were used in 19.3% of presentations. In comparison, emergency transport was required in 27% of cases in a 1-day recreational event (Emond et al. 1999) and ranged from 0.1% of cases (Friedman et al. 1998) to 45% (Boeke et al. 2010) of cases in multi-day recreational events. Our lower percentage of ambulance calls may be due in part to the large number of disposition categories presented. Unique to this study, we were able to present episodes of cyclist medical events that resulted in a wide range of dispositions, including reports where nothing was done (i.e., report only), the rider refused care, or the rider was assessed and continued riding. It is also possible that medical teams present at competitive cycling events allow for more injuries to be treated on site, accounting for the high rate of medical care, and are preventing unnecessary use of emergency medical services. A more thorough investigation of medical team structure is warranted to better understand on-site medical care practices. Future collection of injury race data may benefit from inclusion of data that document treatments and provider type.

Cyclists injured during competition commonly injure more than one body region. In our data, an average of 1.37 injured body regions were recorded per report. Interviews with mountain bike competitors reported as many as 2.75 injured body regions per cyclist (Kronisch et al. 1996). There are a multitude of factors that are different between road cycling and mountain bike which include off-road terrain versus paved roads, proximity to other riders, technical skill required, and equipment used. These differences make comparisons between the two sports difficult and reinforce the need to investigate injuries specific to each discipline of cycling. Medical teams providing care at competitive cycling events must anticipate that cyclists will frequently present with more than one injured body region. Unlike other sporting events where an athlete’s injury can typically be narrowed down to one body region, medical providers at cycling events should be prepared to perform a thorough head-to-toe assessment of the cyclist in order to not miss additional injured body regions.

Results of this study provide an initial look at injury frequencies for each body region in competitive road cycling on a large scale and identify the upper extremity as accounting for 46.5% of all injury reports. This is comparable to previous reports on non-competitive events in which the clavicle was reported as the most frequent fracture (Emond et al. 1999) and accounted for up to 44% of all fractures recorded (Boeke et al. 2010). Over two-thirds of soft tissue injuries reported in non-competitive event occurred in the upper body (Boeke et al. 2010). This is also supported by a frequency of 43% of all injuries occurring in the upper extremity in mountain bike competition (Kronisch et al. 1996). Professional cyclists also reported, through interviews, that injuries to the shoulder girdle and upper extremity accounted for about half of all acute trauma during competition and training (Bernardo et al. 2012). Overall the literature is consistent in reporting the high frequency of upper extremity injuries in cyclists.

The lower extremity was the second most frequently reported injured body part in our data set, at about 32%. This is consistent with reports of injuries in professional cyclists where traumatic injuries occurred in the lower extremity in 26% (Bernardo et al. 2012) and 17.1–23.5% of cases (Barrios et al. 2015). The lower extremity appears to be involved in a much higher proportion, 67%, of overuse injuries (Bernardo et al. 2012) when compared to other body regions. Through the data reviewed, it was not possible to determine whether an injury was acute or overuse, but the potential exists that some racers had existing injuries that were exacerbated in a race and required evaluation from the on-site medical team. Competitive cycling is a lower extremity sport, so it is worth investigating what types of injuries are occurring in the lower extremity and whether these injuries have an effect on the cyclist’s ability to compete.

Head/neck, facial, and back/torso injuries accounted for fewer injury reports in our analysis. However, a much larger proportion of these injured body regions resulted in the use of ambulance/EMS services and referral to doctor/hospital/clinic for additional care. This aligns with findings from an analysis of bicycle injuries that presented to emergency departments (Chen et al. 2013). Medical teams providing care should recognize that while these injuries are not common, they are likely to result in additional medical services and follow-up care for those injured. Therefore, planning to ensure access to emergency services is available and that information can be provided to racers about local hospitals, urgent cares, or clinics may assist in getting injured racers the care they need.

In our study, males accounted for 84.8% of the injury report forms collected. Males account for the majority of participants in non-competitive cycling events ranging from 65% (Boeke et al. 2010) to 68% (Emond et al. 1999) of total participants, and much of what we know about cycling has focused on male cyclists (Barrios et al. 2015; Bernardo et al. 2012; Clarsen et al. 2010). Males and females differed somewhat on disposition categories. Incident reports related to females included more ambulance/EMS transport than males, and males required more “medical attention” than females. In previous reports (Boeke et al. 2010; Emond et al. 1999), males accounted for the majority [57% (Boeke et al. 2010) to 62% (Emond et al. 1999)] of ambulance requests in non-competitive events. Emond et al. (1999) indicated that females, although they made up a lower percentage of the EMS calls, had a higher relative risk (1.3) of requiring EMS transport in single-day non-competitive events. Boeke et al. (2010) presented similar data and found that ambulance transfers for females were significantly higher when accounting for overall participation numbers during a multi-day non-competitive event. In competitive mountain biking, women were 1.94 times more likely to sustain an injury (Kronisch et al. 1996). We do not have exposure data for all cyclists in the races, so it is not possible to calculate relative risks in the current competitive cycling sample. Future research should continue to gather exposure and injury data in competitive cycling to improve ability to compare across variables, such as sex.

The reason for differences in ambulance/EMS utilization between this sample and prior reports is not clear. Injured males in competitive road racing may require more medical care based on a number of factors. If the competitive field sizes are larger for males and more riders are being forced onto a racecourse at the same time, there would be a potential increase in the risk of injury to those racers. Since males also race at higher average speeds (Abbiss et al. 2013), this may result in higher velocity crashes. A future analysis that includes the total number of male and female cyclists in the events would allow for a more complete analysis of whether sex is associated with risk for medical attention.

We also explored age as it relates to injury disposition. Results suggest that, for the most part, age did not impact the disposition following injury. The only difference noted was between released to parent/personal vehicle and refused care, with those refusing care being older than those who were treated and released. Previous studies (Barrios et al. 2015; Clarsen et al. 2010) examining injuries in competitive cyclists only examined participants with an average age in the mid-20s. Additionally, a study related to cyclists who presented to emergency departments indicated that those cyclists were largely under 34 years of age (Chen et al. 2013). Our study included a more diverse age range of participants and reported on medical events associated with cycling that are typically unreported when data are captured through emergency department records. Medical teams may be acting more cautiously when examining and documenting care provided to minors, and this may partly explain the large number of participants in the “release to personal vehicle/parents” category. The relationship between competitive cyclist age and factors leading to receiving or refusing care, particularly the care of pediatric-aged athletes, should be further investigated.

This study should be interpreted in the context of several limitations. First, data from this analysis should only be generalized to competitive road cycling events. Just as data from non-competitive events or mountain bike events do not lend itself to competitive road cycling, data from this analysis should be interpreted similarly. Data regarding exposures, such as the number of events in each state examined, the total number of cyclists in each event, the distance and time spent in competition, and the total number of males and females participating in each event were not available for analysis which limits the ability to determine rates of injuries. However, the large number of incident reports and the fact that these data were captured across 30 states provide a strong foundation of data to draw initial description and insight into the types of injuries suffered by competitive road cyclists. Given that we analyzed all injuries that were reported, and not just those that required EMS/ambulance or hospital care, there is the potential that we are over-estimating the number of meaningful medical events. Reporting on number of injuries per cyclist, injuries per distance traveled, or time spent competing is common in the literature (Boeke et al. 2010; Emond et al. 1999; Roi and Tinti 2014; Barrios et al. 2015; Bernardo et al. 2012) and should be emphasized in future injury surveillance efforts. In order to present more complete information on incidence rates, more demographic information captured in a consistent manner is needed. A summary sheet that accompanies race incident reports and provides an overview of the race (e.g., number of racers, number males/females, distance traveled, weather conditions) is a recommendation to enhance prospective cycling injury surveillance. Another limitation to the data includes the incomplete nature of information provided on some of the incident report forms. Sex and age were not recorded in the injury reports for a number of cyclists, which shrinks the pool of data to use for analysis. Lastly, the data were collected and recorded solely by the lead author, so there may be minor errors present in the data set. Despite these limitations, having data prospectively collected by personnel at each competitive event provides a more complete report of injuries than relying on patient self-reports or emergency department data.

## Conclusions

Cyclists participating in competitive road cycling events most commonly use on-site medical services, but a substantial percentage require EMS transport. Injuries to females and injuries involving the head, neck, face, and torso resulted in the largest proportions of emergency medical service usage. Medical teams providing care at competitive road cycling events should anticipate encountering injuries to the upper extremity and expect that riders may suffer injuries to more than one body region. Additionally, because emergency transport is a real possibility during a race, efforts to ensure the transport procedures are in place and practiced prior to an event are suggested to promote positive outcomes for injured racers. More research gathering detailed epidemiology data, such as injury rates and risk ratios, and day of event information, such as total number of cyclists, distance, or time spent in competition, are needed to advance the understanding of injuries in competitive cyclists and to identify ways to reduce injuries during road racing events.

## Data Availability

The datasets used and/or analyzed during the current study are not available publicly; however, deidentified datasets are available from the corresponding author on reasonable request.
